# Assessing future technological impacts of patents based on the classification algorithms in machine learning: The case of electric vehicle domain

**DOI:** 10.1371/journal.pone.0278523

**Published:** 2022-12-06

**Authors:** Fang Han, Shengtai Zhang, Junpeng Yuan, Li Wang

**Affiliations:** 1 National Science Library, Chinese Academy of Sciences, Beijing, China; 2 School of Economics and Management, Beijing University of Posts and Telecommunications, Beijing, China; 3 Department of Physics, Beijing Normal University, Beijing, China; Southwest Jiaotong University, CHINA

## Abstract

**Introduction:**

Identifying the technologies that will drive technological changes over the coming years is important for the optimal allocation of firms’ R&D resources and the deployment of innovation strategies. The citation frequency of a patent is widely recognized as representative of the patent’s value. Thus, identifying potential highly cited patents is an important goal. A number of studies have attempted to distinguish highly cited patents from others based on statistical models, but a more effective and applicable method needs to be further developed.

**Methods:**

This paper treats the prediction of later patent citations as a classification problem and proposes a novel framework based on machine learning methods. First, a indices system to identify highly cited patents is constructed using multiple factors that are believed to influence citation frequency. Second, various machine learning models are utilized to identify highly cited patents. The optimized model with the best generalization capability is selected to predict the future impacts of newly applied patents, which may be representative of emerging significant technologies. Finally, we select the electric vehicle (EV) domain as a case study to empirically test the validity of this framework.

**Results:**

The optimized support vector machine (SVM) model performs well in identifying highly cited EV patents. Technological frontiers in the EV domain are identified, which are related to the topics of information systems, batteries, stability control, wireless charging, and vehicle operation.

**Discussion:**

The good performance in prediction accuracy and generalization capability of the method proposed in this paper verifies its effectiveness and feasibility.

## 1 Introduction

The anticipation and forecasting of technological changes are important because technological advances have become increasingly fast and complex. The core issue is the identification of current technologies that will drive technological changes over the coming years [1]. While a patent’s citation frequency is widely accepted as one important measure of the importance of the patent in a particular technological area [2–5], predicting the citation frequency of new applied patents in the coming years is helpful to predict upcoming technologies that will be critical to firms and governments in the current competitive environment. However, it generally requires a long time to reach the citation peak after the publication of patents [6], so previous studies that have explored key innovations using citation frequency could not predict forthcoming critical technologies. In other words, predicting the citation frequency of patents or grasping features that affect this frequency is helpful to explore potentially important technologies. Newer techniques also emphasize the importance of citation centrality in assessing potential progress [7, 8].

**Fig 1:**
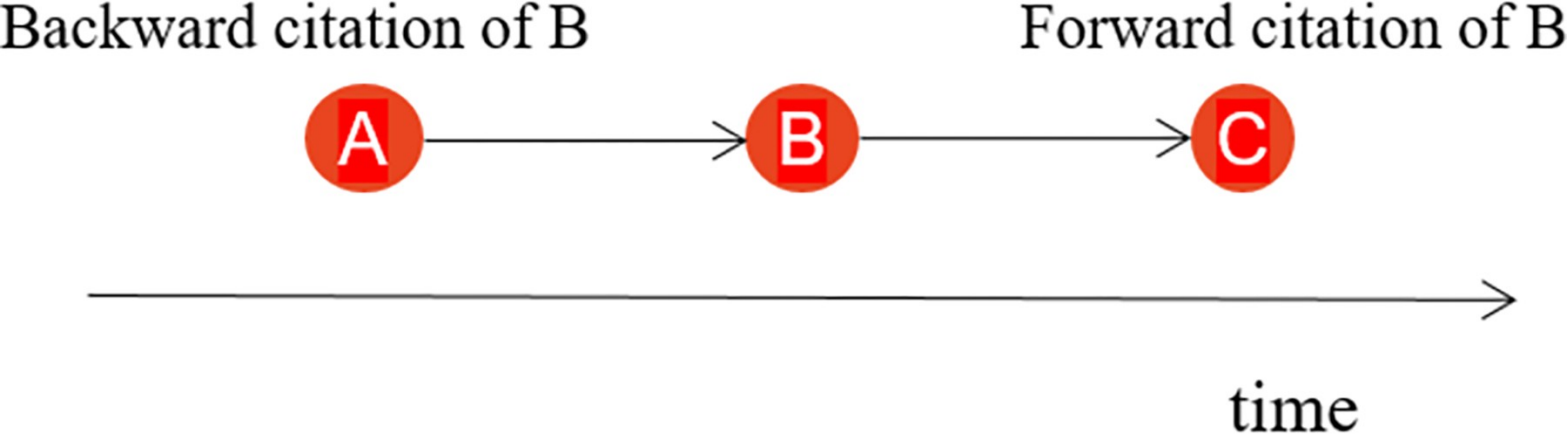
Illustration of “backward citation” and “forward citation”. A, B, and C are three patents. If A was cited by B and B was cited by C, then A was the backward citation of B and C was the forward citation of B.

Compared with citation prediction research on academic papers [9, 10], relatively few studies have explored the features of highly cited patents, and most researchers focus on citation analysis rather than citation prediction [11]. Nevertheless, some statistical models have been established to identify the features of highly cited patents and predict future citation behavior. Lee et al. proposed a stochastic patent citation analysis to assess future impacts over a particular period [1]. Yoshikane et al. [12] confirmed the significant positive correlation between the number of citations a patent receives and the number of the cited patent’s classifications. Yoo and Chung [13] applied a multiple regression model to distinguish important factors based on 17 explanatory variables in five subject fields. Their research showed that the number of pages, number of IPC codes of backward citations (i.e., cited patents; see Fig 1), number of claims, and four other indicators have impacts on the citation frequency of patents. The variables in different fields showed significant differences. With a focus on Korean medical device patents, Yoon et al. [14] used 13 technical indicators to develop a generalized linear model of the number of patent citations and found 7 influential indicators, including the number of inventors, countries for priority claims, and the number of IPC codes. Lin et al. [6] predicted citations of biotechnology patents based on regression analysis.

These studies have shed light on the impact factors that are correlated with citation frequency. However, most studies have not comprehensively considered multiple factors. For instance, even though many previous studies have demonstrated the close relationship between science and technology [15, 16], studies have not adopted science-related indices, such as the number of scientific citations, the diversity of the cited scientific categories, and other variables linking science and technology, to the influencing factors. Furthermore, most previous studies have explained and predicted patent citation frequency based on regression analysis. However, patent citations often have obvious long-tail effects, make such predictions necessarily skewed. Thus, it is not suitable to use a regression model in studies [17]. Given this background, this study considers a broader scope of factors that may affect citation frequency. And we regard the prediction of citation frequency as a classification issue and calculate the contribution of each variable based on different machine learning methods. The optimized model with the best performance is selected to predict potential highly cited patents. Compared with the regression method, the classification algorithm used in this paper has much better performance.

This paper is organized as follows. First, we describe the source data used in the analyses and explicate the methodology, including the variables representing the quantitative characteristics of patents and the machine learning models. Second, we present the results of the analyses, in which the optimized model is selected and the contributions of the factors to citation frequency are calculated based on the model and are used to predict potential highly cited patents. We use the EV domain as a case to illustrate this method and verify its effectiveness. The last section concludes.

## 2 Materials and methods

### 2.1 Data

The patent data were downloaded from the patent search tool PatSnap, and keywords related to the research domain were searched in US patents. The obtained data included the patent number, associated classifications, numbers of inventors, number of claims, and other useful information that may contribute to the citation frequency. The citation frequency within 3 years could also be obtained directly from PatSnap.

### 2.2 Index system of highly cited patents

The response variable in this paper is the citation frequency within 3 years of the patent (*Fcited*), which is proven to be representative of the core technology [18]. And our discussion in section 3.2 also proved that this indicator could be utilized as the measurement of patent impact in the EV domain.

Combining previous studies and the principles of comprehensiveness, measurability, and availability once published, 17 explanatory variables, including the characteristics of patents, knowledge source, and inventors, which have been recognized as commonly influencing citation frequency, were adopted into the analysis as follows.

#### 2.2.1 Characteristics of patents

Number of associated classifications (*VC*): the number of International Patent Classification (IPC) codes attributed to the patent, which can indicate the breadth of technological knowledge of the patent. It has been proven to be positively related to citation frequency [11]. A previous study noted that examining the lower-level classifications could produce a higher value of the correlation coefficient with the citation frequency [1]. Therefore, this study utilizes the number of IPC codes at the bottom level in the IPC.

Number of new associated classifications (*VCnew*): the number of IPC codes that first appeared in the domain. Previous studies have shown a correlation between innovation and new technological knowledge [19, 20]. Thus, in this paper, we use the new IPC codes (at the bottom level) to represent the new knowledge of the patent.

Number of new combinations of associated classifications (*CCnew*): the number of new combinations of IPC codes (at the bottom level) in the patent. Based on technological convergence theory, the fusion of different technological categories can lead to disruptive technology [21, 22]. Thus, this index is expected to be correlated with citation frequency.

Number of patents in patent family (*FM*): a commonly used index in the assessment of patent value. A previous study showed that patents representing large international patent families are particularly valuable [23].

In addition, referring to previous studies, this paper adopts the number of figures (*FG*), number of pages (*PG*) and number of claims (*CL*), which can represent quantities of descriptions or can be specific to patent applications, as the explanatory variables [11].

#### 2.2.2 Characteristics of the knowledge source

The knowledge source can be divided into two aspects: technological knowledge from backward citations and scientific knowledge from cited papers. The related indices we selected are as follows.

Number of backward patent citations (*VTciting*): previous studies have found that highly cited patents have more citations than others [24]. This could be because inventing is a recombinant process, and the value of invention is associated with how many prior inventions are considered [25]. Thus, this study takes the number of backward citations as the explanatory variable.

Number of associated classifications with backward patent citations (*TCciting*): this indicator reflects the diversity of technological knowledge sources. Many researchers have found that technological diversity is correlated with the novelty of patents, which is related to citation frequency. Examples include Rosenkopf and Nerkar [19] and Yoshikane et al. [12]. Their studies showed that breakthrough inventions often cite more classifications than others. Based on these studies, this study calculates the number of citing classifications at the subclass level (the fourth layer in IPC).

Novelty of backward patent citations (*NTciting*): A previous study showed that the novelty of backward citations is related to patent value [26]. Thus, if a patent cites more newly published patents, it seems to be cited more frequently. We adopt the proportion of newly published patents in 5 years as the measure of the novelty of the technological knowledge source.

The relationship between scientific knowledge development and technological development is widely recognized as one of the most important and complex aspects of technological evolution. Gittelman and Kogut [27] established that the number of scientific citations is correlated to technological innovation. Han and Magee [28] confirmed that the structure of scientific citations, which has been measured by scientific categories, is related to the technological structure. Scientific references in patents can be added by both the applicants and the examiners [29]. In this paper, the scientific citations of both sources are considered to have the same function of linking science with technology. Since scientific citations represent a bridge between science and technology, they do not necessarily indicate the direct scientific basis of the patent [30]. We adopt the number of nonpatent literature (*NPL*), number of scientific citations (*VSciting*), number of categories associated with the scientific citations (*SCciting*), and novelty of scientific citations (*NSciting*) as the quantities specific to the scientific knowledge source. In this paper, the scientific citations are papers indexed by Web of Science, which are identified using the MATLAB tool. The classifications of journals by Journal Citation Report (JCR) are utilized in this paper to define the scientific categories.

#### 2.2.3 Characteristics of inventors

Number of inventors (*IV*): A previous study found that the number of inventors has a positive relation with citation frequency in most IPC sections [9]. This indicator can reflect the R&D input and the breadth of knowledge of the inventors to some degree.

In addition, the average number of patents of all inventors (*VPaverage*) is utilized as the explanatory variable. Many studies have shown the important role of personal capabilities on patent value [31, 32]. Therefore, in this paper, the average number of patents in a domain of all inventors and the number of patents of the first inventor (*VPfirst*) are used to measure personal capability.

Table 1 details the procedures employed to derive these indices from the data.

**Table 1:** Derivation procedure for each index.

Dimension	Index	Procedure of derivation
Patent	Number of associated classifications (*VC*)	Counting the number of associated classifications at the group level
	Number of new associated classifications (*VCnew*)	Counting the number of first time appeared associated classifications at the group level
	Number of new combinations of associated classifications (*CCnew*)	Counting the number of new combinations of associated classifications at the group level
	Number of patents in patent family (*FM*)	Extracting the value from the field of the "simple family count"
	Number of figures (*FG*)	Crawling the value of "figures" for each patent from Patsnap website
	Number of pages (*PG*)	Extracting the value from the field of the "count of pages"
	Number of claims (*CL*)	Extracting the value from the field of the "count of claims"
Knowledge source	Number of backward patent citations (*VTciting*)	Extracting the value from the field of the "count of cites patents"
	Number of associated classifications with backward patent citations (*TCciting*)	Extracting all classifications for each backward patent citation and counting the number of different classifications at the subclass level
	Novelty of backward patent citations (*NTciting*)	Calculating the percentage of backward patent citations published in 5 years
	Number of nonpatent literature (*NPL*)	Extracting the value from the field of the "count of NPL"
	Number of scientific citations (*VSciting*)	Counting the number of scientific citations indexed by WoS
	Number of associated categories with the scientific citations (*SCciting*)	Counting the number of scientific citation categories classified by JCR
	Novelty of scientific citations (*NSciting*)	1/(published year of the patent-average published year of all backward scientific citations)
Inventors	Number of inventors (*IV*)	Extracting the value from the field of the "count of inventors"
	Average number of patents of all inventors (*VPaverage*)	Counting the number of patents invented by all the inventors of the patent divided by the number of inventors
	Number of patents of the first inventor (*VPfirst*_)_	Counting the number of patents invented by the first inventor of the patent

### 2.3 Machine learning model building

In this paper, patent citation prediction is regarded as a classification problem. There are many alternative methods to use machine learning for classification and prediction. This paper selects three classic machine learning classification methods that have been successfully applied in various fields: support vector machine (SVM), random forest (RF), and extreme gradient lifting (XGBoost).

#### 2.3.1 SVM

SVM is a data classification technique developed by Cortes et al. [33] according to supervised learning. First, it can only solve the problem of binary classification. However, most of the practical applications are multiclassification problems, so two methods are designed: "one-against-one" and "one-against-the-rest". The SVM method has a strong theoretical basis, and the extremum solution obtained is the global optimal solution, so the SVM model has good generalization ability for unknown samples and has been widely used in various fields.

#### 2.3.2 RF

RF was proved and completed by Breiman in 2001 [34], is a classifier containing multiple decision trees. RF selects several groups of mutually independent classifiers to form the final classifier structure and then averages the output results of each decision tree to obtain the final classification result. RF has the advantages of processing unbalanced datasets, effectively reducing the probability of overfitting and fast training speed.

#### 2.3.3 XGBoost

XGBoost was proposed by Chen and Guestrin [35], is based on the boosting idea. It is a supervised and extensible tree lifting algorithm derived from the gradient lifting algorithm. In the process of iterative optimization, the XGBoost algorithm performs second-order Teller expansion on its loss function, so it can estimate the loss function more accurately. In addition, the XGBoost algorithm has the advantages of effectively dealing with missing values, preventing overfitting, and improving the computing speed.

**Fig 2:**
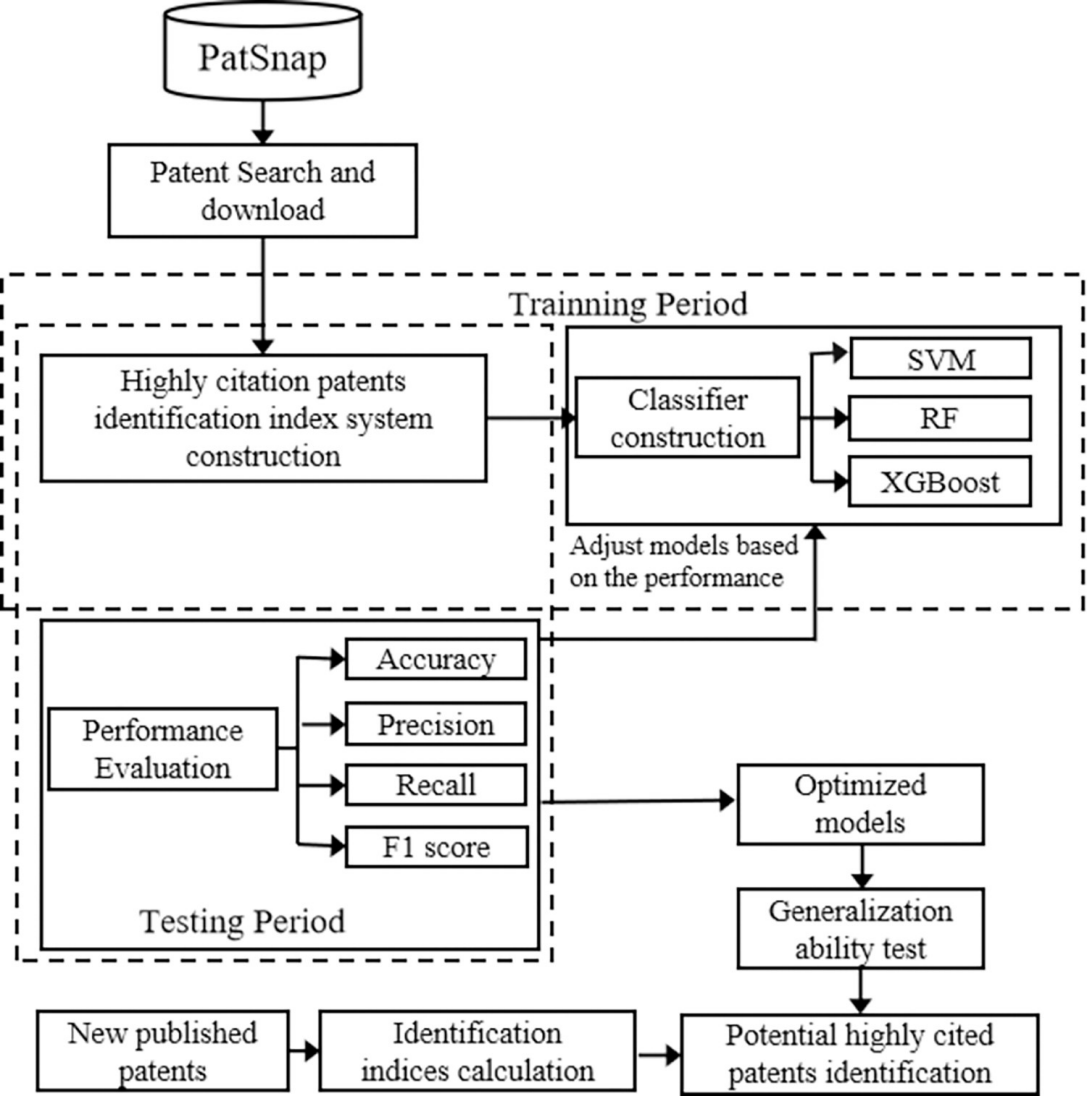
Potential highly cited patent identification model based on machine learning.

The accuracy rate, precision rate, recall rate, and F1 score [36] are used to evaluate the performance of the models in this paper. The influences on citation frequency are calculated for each explanatory variable through the optimized machine learning model with the best generalization ability, which can be further utilized to predict potential highly cited patents based on the newly published patents. The frame of the structure is as follows (Fig 2).

## 3 Empirical analysis

### 3.1 Data

This study takes the electric vehicle domain as an example to verify the validity of the identification model we proposed. We searched the patent data using the keywords “electric vehicle” and “electric car” by utilizing the patent search tool PatSnap, which searched 13732 US patents from 1976 to 2021 (the EV patent data can be downloaded from S1 File). Since the response variable is the citation frequency in 3 years, we selected the patents that were published between 2000 and 2016, which include 5665 patents whose citation numbers are already known, to study the influences of the explanatory variables. We selected top 20% patents ranked by citation frequency in 3 years as the highly cited patents. At last, 1149 with a citation frequency ≥10 are selected as the highly cited patents and used to test the performance of various machine learning models after classification.

### 3.2 Construction of the highly cited patent identification indicator system

**Fig 3:**
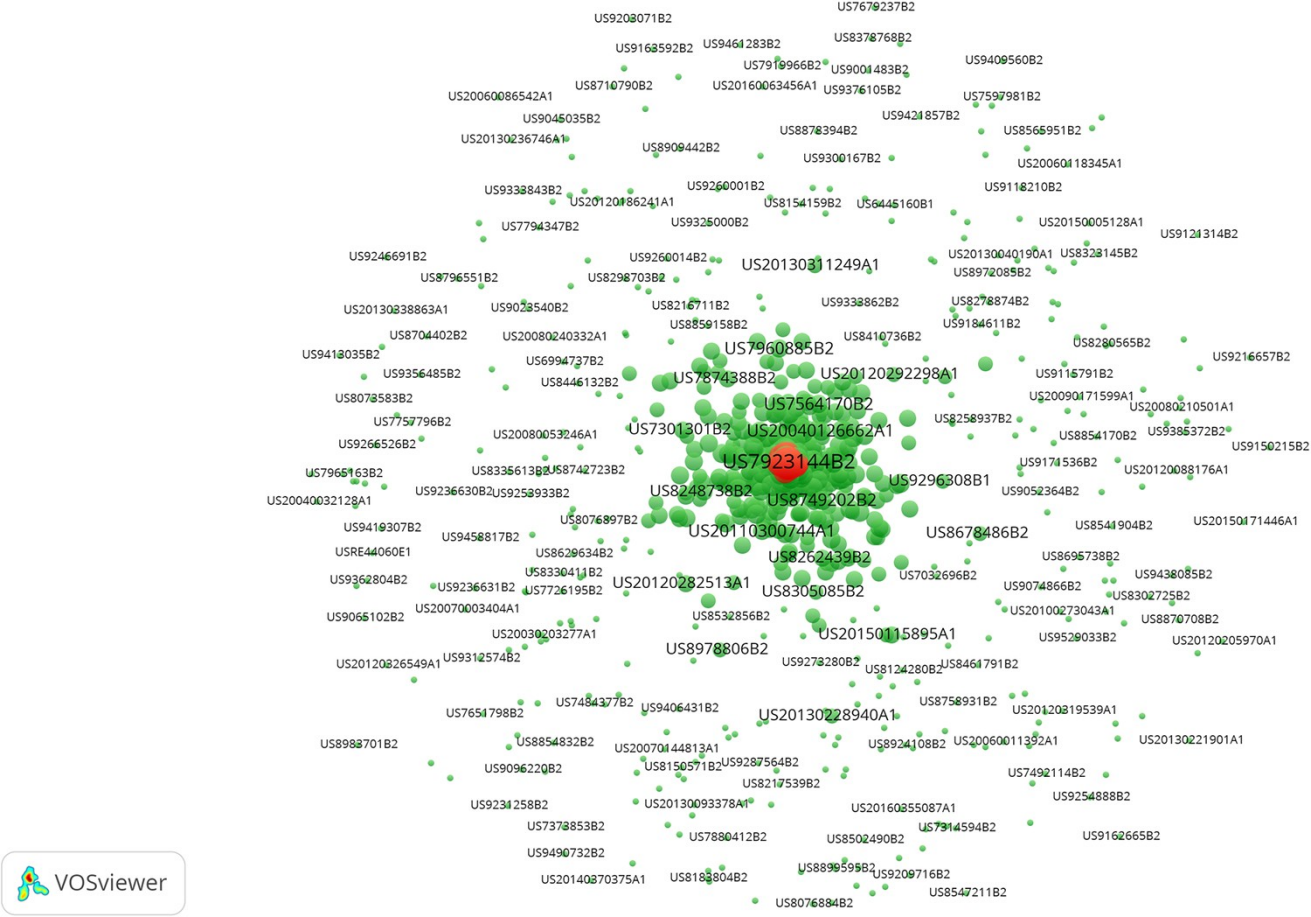
The citation-base map of the EV patents. The highly cited in 3 years are marked in red, and the non-highly cited ones are marked in green.

**Fig 4:**
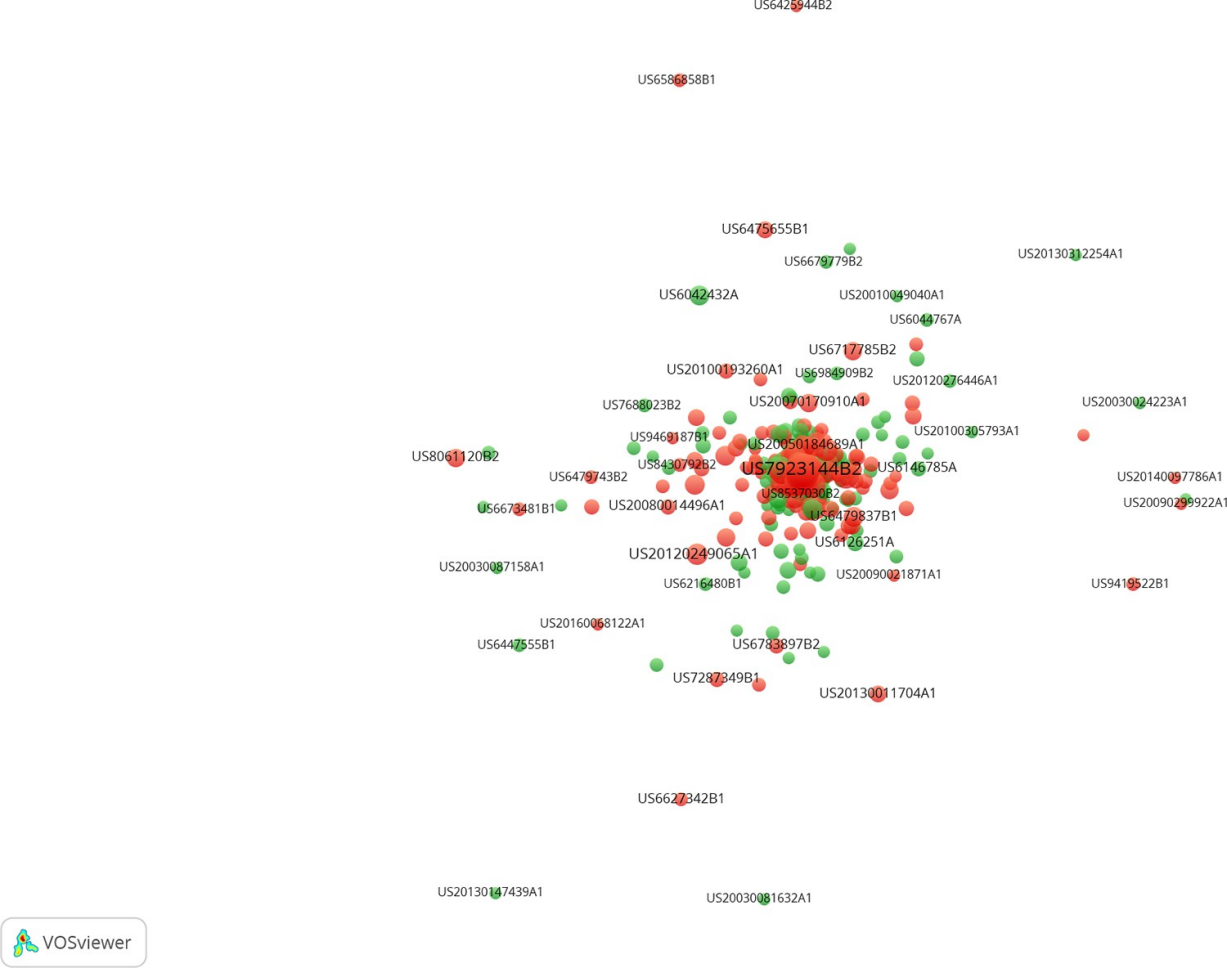
The central part of citation-base map of the EV patents. The highly cited in 3 years are marked in red, and the non-highly cited ones are marked in green.

**Fig 5:**
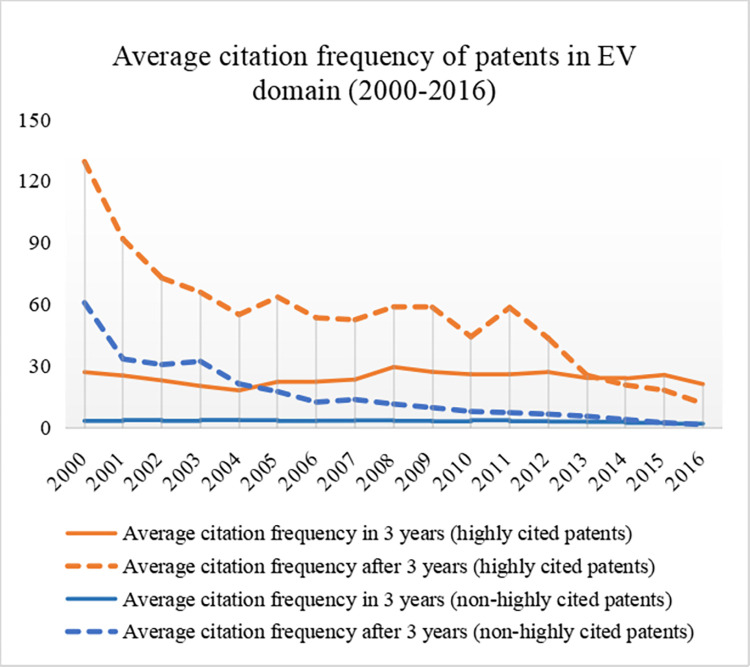
The average citation frequency of highly cited patents and non-highly cited patents in 2000–2016.

As mentioned above, this study utilizes the indicator of citation frequency in 3 years of the patent as the response variable. To show the citation relationships in the EV domain and verify the feasibility of the selected indicator, we applied Gephi [37] and VosViewer [38] to generate the citation-based map of the EV patents based on their citation relations with all other patents from 2000 to 2021. For better visualization, we display only the 5665 EV patents published between 2000 and 2016 (Fig 3). The 1149 highly cited patents are marked in red, and the non-highly cited patents are marked in green. Fig 4 shows the core part of the map. The maps show that the patents with high citation frequency in 3 years (the red nodes) hold the central position in the whole period. This means that they acquire more citations than the others. Fig 5 further supports this viewpoint: the highly cited patents (in 3 years) still acquire more citations than the others published in the same year after 3 years. The Pearson correlation coefficient is 0.61 between the citation frequency in 3 years and the citation frequency after 3 years for all EV patents published between 2000 and 2016, which shows a relatively strong relationship between them. This result also proves that the indictor of citation frequency in 3 years could be utilized as the agent of future impact of the patent in the EV domain.

We defined three patent classifications based on the number of forward citations in 3 years, which could be representative of the high impacts at different levels (patents with a C2 class are the most valuable, C1 class patents are the “mediumly high” valuable, and C0 class patents are the “normally high” valuable). Considering the balance and feasibility of the data requirements, the median of the cited frequency data in each class is used to decide the threshold values. The difference between the medians in C0 and C1, should be approximately equal to the difference between the medians in C1 and C2. Table 2 shows the detailed data classification criteria.

**Table 2:** Data classification criteria.

	C0	C1	C2
Fcited	[10, 40]	[41, 62]	≥63
Median	15	48	81.5
Number of patents	997	86	66

After classification, we discussed the relationships between explanatory variables and between each of these and the response variable based on Pearson correlation test. Table 3 shows the part of results. Strong correlations are observed for 4 pairs variables, including *VC* and *CCnew*, *FG* and *PG*, *VTciting* and *TCciting*, *VPaverage* and *VPfirst*. Considering the multicollinearity between variables judged from the observed values of the correlation coefficients, we included only one variable in each pair of them in the following analyses. *VC*, *PG*, *TCciting* and *VPaverage* are selected because they show slightly stronger correlations with the response variable. Thus we obtained 13 explanatory variables.

**Table 3:** Correlations between variables.

	*VC*	*VCnew*	*CCnew*	*FM*	*FG*	*PG*	*CL*	.* *.* *.	*Fcited*
***VC***	-	0.470	0.817	0.143	-0.009	-0.003	0.002	. . .	-0.043
***VCnew***	0.470	-	0.618	0.086	0.017	0.037	0.065	. . .	0.040
***CCnew***	0.817	0.618	-	0.129	0.010	0.012	-0.002	. . .	0.000
***FM***	0.143	0.086	0.129	-	-0.033	-0.059	0.131	. . .	-0.051
***FG***	-0.009	0.017	0.010	-0.033	-	0.931	0.178	. . .	0.198
***PG***	-0.003	0.037	0.012	-0.059	0.931	-	0.186	. . .	0.238
***CL***	0.002	0.065	-0.002	0.131	0.178	0.186	-	. . .	0.135
**.* *.* *.**	. . .	. . .	. . .	. . .	. . .	. . .	. . .	. . .	. . .
***Fcited***	-0.043	0.040	0.000	-0.051	0.198	0.238	0.135	. . .	-

**Fig 6:**
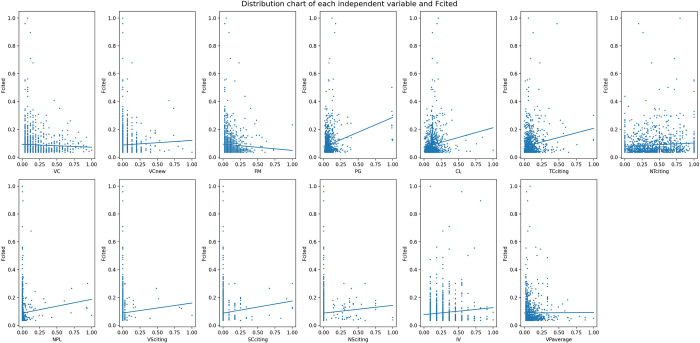
Relationships between each of the explanatory variables and *Fcited*.

In general, all explanatory variables except *VC and FM* have positive values of the Pearson correlation coefficients with *Fcited*. And compared with the other variables, *PG* and *FG* show relatively strong correlations with *Fcited*. These are followed by *TCciting* and *CL*. However, correlations between the explanatory variables and *Fcited* are generally weak. Most of the absolute values of the correlation coefficient with *Fcited* are less than 0.20. Fig 6 visualizes the relationships between each of the 13 explanatory variables and *Fcited*.

At last, we obtained the highly cited patent identification indicator system of the EV domain (Table 4), which is a 1149×15 matrix. The first column is the publication number (PN) of the patents, Columns 2 to 14 are 13 explanatory variables used as input data for machine learning models, and the last column shows the classifications of the patents, which are the output data for the machine learning models.

**Table 4:** An example of the highly cited patent identification indicator system.

	*VC*	*VCnew*	*FM*	*PG*	*CL*	.* *.* *.	*Classification*
US20140191568A1	2	0	2	36	34	. . .	C2
US9026347B2	3	0	4	47	20	. . .	C2
US8076801B2	1	1	23	67	67	. . .	C2
US20110298427A1	10	0	3	15	7	. . .	C1
US20120112536A1	2	0	1	292	18	. . .	C1

### 3.3 Comparison of the performance of machine learning models

Based on the SVM, RF, and XGBoost models, we randomly selected 80% of the data for training, and the other 20% of the data are utilized for performance testing. The parameters of each model are optimized based on their performance, and the accuracy, precision, recall, and F1 score are used to measure the performance of the models. Table 5 shows that all the measurement indices of the optimized models are greater than 0.75, which indicates the validity and feasibility of our method.

**Table 5:** Classification performance of each machine learning model.

Model	Accuracy	Precision	Recall	F1
SVM	0.861	0.770	0.861	0.802
RF	0.852	0.799	0.852	0.806
XGBoost	0.839	0.751	0.839	0.791

**Fig 7:**
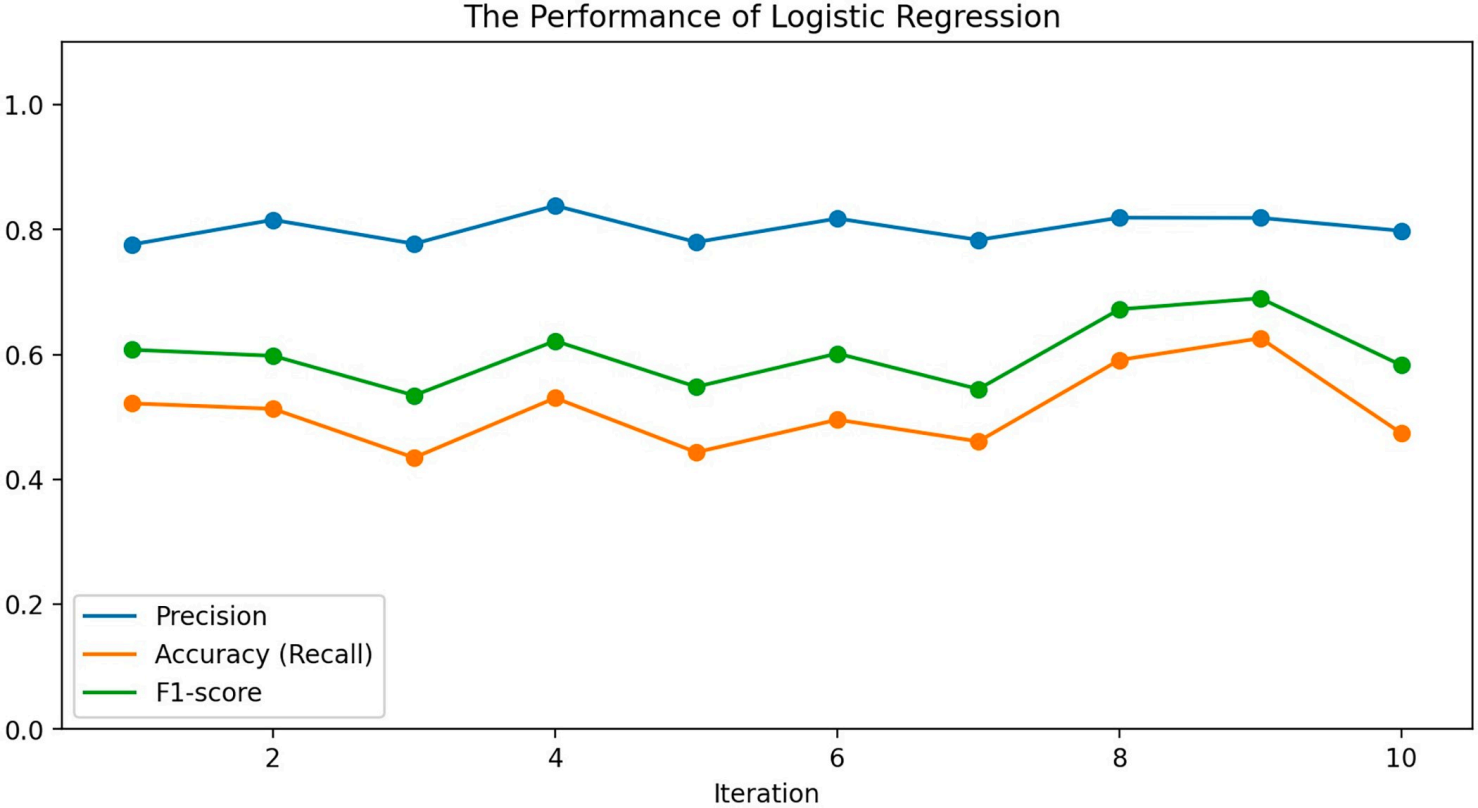
The performance of logistic regression model.

To compare with the previous methods, this study also conducts the regression models, including the multiple linear regression and multiple logistic regression, which have been widely used in the previous studies [6, 12–14]. Results show that the linear regression is not statistically significant, indicating that it does not fit the data well. And the performance of logistic regression model (optimized by Bayesian optimization parameter adjustment) is relatively poor compared to the models used in this study. Fig 7 shows its performance under the 10-folds cross validation. The results further suggest that the regression models are not suitable for the citation prediction [17].

### 3.4 Identification of potential highly cited patents

#### 3.4.1 The generalization ability of the machine learning models

This paper utilizes patents published in 2017 and 2018 to verify the generalization ability of the machine learning models. According to the methods utilized in Section 3.2, the index system for the 1840 patents is constructed and applied to the various machine learning models to predict their citation frequency after 3 years. Compared with the actual data, the accuracy of the three models of SVM, RF, and XGBoost is 0.998, 0.997, and 0.986, respectively, which further supports the feasibility of our method. The SVM model, with the best generalization ability, is used to construct the index system of highly cited patents (Table 6) and predict potential highly cited patents published from 2019 to 2022, which are representative of the technological frontier of this domain.

**Table 6:** The index system of highly cited patents in the electric vehicle domain based on the SVM model.

Index	Weight
Number of pages (*PG*)	0.1511
Number of associated classifications with backward patent citations (*TCciting*)	0.1408
Number of claims (*CL*)	0.1267
Novelty of backward patent citations (*NTciting*)	0.1246
Average number of patents of all inventors (*VPaverage*)	0.1046
Number of patents in patent family (*FM*)	0.0933
Number of associated classifications (*VC*)	0.0767
Number of inventors (*IV*)	0.0671
Number of new associated classifications (*VCnew*)	0.0425
Number of nonpatent literature (*NPL*)	0.0303
Novelty of scientific citations (*NSciting*)	0.0167
Number of associated categories with scientific citations (*SCciting*)	0.0137
Number of scientific citations (*VSciting*)	0.0119

Table 6 shows that the indices that represent quantities of descriptions in a patent document, such as the number of pages and the number of claims, have the most significant influences on the citation frequency. And compared with the characteristics of the patent (such as the number of new classifications), the indicators that are representative of the characteristics of technological knowledge sources (e.g., the number of associated classifications with backward patent citations and the novelty of backward patent citations) contribute more to the citation frequency. The results are basically consistent with those of Yoshikane [11]. They also agree with the features of highly cited scientific papers. The number of backward citations of papers has been found to be strongly correlated to the citation frequency. For example, Kostoff found that highly cited papers in the journal Lancet have a 33% to 300% greater average number of references per article than those that are less frequently cited [39]. Snizek et al. found a strong relationship between the number of backward citations and forward citations for papers in the DNA domain [40].

It is also interesting to note that in contrast to the important effect of the reputation of the author on the citation frequency of a paper [41], the characteristic indices related to the “inventor” only show moderate effects on the citation frequency of patents. There are 2339 inventors who have highly cited patents from 2000 to 2016 in this study, but only 71 of them have earlier patents published before 2000 in the EV domain. Thus, most of the inventors of highly cited patents between 2000 and 2016 are not “famous” people. However, after comparing the citation frequency (within 3 years) between the 71 inventors’ 133 earlier patents and all earlier patents, we found that the 71 inventors’ patents tended to be cited more than the others on average: 36.84% of the earlier patents by the 71 inventors were highly cited patents (cited frequency≥10), and 19.30% were in the whole set. These results show that there is a relationship between the reputation of the inventors and the citation frequency, but the relationship is not as close as in scientific papers.

Finally, the scientific source indices, which include the number of scientific citations and the number of scientific categories, show weak relationships with the citation frequency. This might be because patents rarely cite scientific papers. For example, in this study, only 392 patents cite 1732 scientific papers. In addition, a previous study showed that references to nonpatent literature (*NPL*) are only informative about the value of pharmaceutical and chemical patents but not other technical fields [23].

#### 3.4.2 Potential highly cited patents and frontier technologies

Based on the optimized SVM model, we predict the potential highly cited patents by applying a total of 4522 newly published patents in the years from 2019 to 2021. A total of 245 potential highly cited patents are identified, and the related topics are extracted using the LDA model and the extraction of terms (using the Cortext) based on the titles and abstracts of the patents. Jointly considering the coherence score [42] and the volumes of data, the optimized number of topics is set to 5. The detailed keywords and topics are summarized in Table 7.

**Table 7:** Technological frontier topics in the EV domain.

ID	Topics	High frequency terms (Part)
1	Battery	battery pack|charging station|acid battery|recharge battery|porous membrane|charging status|fast charging|hybrid electric vehicle|layer containing magnesium|active material layer
2	Intelligent information management	cloud server|configuration information storage|data production|third party application|user account|RF signals|gaze detection|user interface|communication APIs|transaction scheduling algorithm
3	Stability control	drive train coolant|drive axles|hybridization strategy|suspension systems|control system|silica-based aerogel|tire pressure detection|flame-resistance|plurality of sensors|tire state
4	Wireless charging	charge transactions|peer-to-peer|magnetic coupling|computer program code|determining charge levels|wireless power receiver|contact pins|distributed generators|contactless communications|FOD sense loops
5	Vehicle operation	graphene oxide|power management|magnetic field|more parking spaces|brake operation|rotatable vehicle drive|temperature sensor|tracking device|OBD data|vehicle in response

The battery topic covers the battery framework design, battery materials, battery charging, etc. The battery pack is the most challenging item in terms of reliability and safety issues of EVs, so the protection of batteries is of great importance [43]. The utilized magnesium in the EV battery is also noteworthy. Compared to lithium, magnesium has some advantages, including low cost and high energy density. Thus, magnesium-lithium hybrid batteries are regarded as the most promising technologies in the EV battery domain [44]. The charging technologies include fast charging and charging connectors. Modifying the charging connector could cut off the power if there is an overheating issue in the battery and to avoid fire, so it is vital to the EV [45]. Fast charging is important for the current implementation of electric mobility systems and is directly related to the user experience. Improving the quality of the energy transferred by charging stations to the vehicle requires further innovation.

Intelligent information management involves the technologies of cloud systems, configuration information storage, data production, third-party applications, etc. An intelligent and connected vehicle (ICV) cloud system is regarded as a potentially synthetic solution for high-level automated driving to improve safety and optimize traffic flow in intelligent transportation [46]. Among the various technologies, a third-party application is noticeable; it can be used for the exchange of information with cloud-based processing systems and for managing user profiles for vehicles, and it is helpful to protect data privacy and secure transactions [47].

Stability control is related to insulated and fire-retardant, tire state detection, and braking technologies. The operation of an electric vehicle produces a significant amount of heat, which requires coolant to help control temperature and guarantee safe operation [45]. In addition, the silica-based aerogel has attracted a great deal of attention due to its lightweight and stable insulation performance [48]. The tire state of EVs is crucial to vehicle stability and driving safety. Related technologies include tire pressure detection and other technologies.

Wireless charging is a challenging issue in the domain of EVs. It includes the magnetic coupling design, charge monitoring, and power transmission technologies. It has the advantages of convenience and high speed and is expected to have bright development prospects. Magnetic field coupling has high reliability, flexibility, and security and has been successfully used in wireless charging in several domains [49]. However, the requirement of high transmission efficiency in the EV domain requires more innovation in the future. The charge level monitoring is directly related to the driver’s safety. Foreign object detection (FOD) is significant in wireless charging to prevent the system from overheating [50].

Vehicle operation topics include the technologies of power management, parking space detection, brake operation, and reaction operation. Due to the large current, the graphene cell can be charged quickly and has excellent future prospects [51]. With the growth in EV sales, the demand for parking at charging points has vastly increased. Thus, intelligent parking management has attracted considerable attention from manufacturers [52]. In addition, the sensing system and on-board diagnostic (OBD) system are vital to the driving safety and performance maintenance of EVs. For example, the user can be quickly identified through the OBD data [53]. However, the OBD technology applied in EVs is imperfect and should be improved in the future [54].

## 4 Conclusion

Early identification of the technological frontier is important for the optimal allocation of enterprises’ R&D resources and the formulation of government innovation strategies. Many scholars have used bibliometric methods to identify the technological frontier, and the citation analysis method has been widely used. However, it takes a certain amount of time to accumulate citations of patents. The existing citation analysis method cannot incorporate newly published patents, which are potentially highly cited, into the data collection of the important patents used to identify the technological frontier. Therefore, this paper proposes a novel framework for identifying the technological frontier by utilizing classification algorithms based on machine learning models. The electric vehicle domain is used to verify the availability of our method.

Compared with the multiple regression model used in previous studies, the classification algorithm based on machine learning models used in our study has many advantages. First, the citation frequency of patents often shows a long-tail effect, which is not suitable for a regression model. Second, coarse-grained models perform better in fitting accuracy and have better generalization abilities. In addition, this study considers a broader scope of factors that may affect citation frequency than previous studies, which could increase the accuracy of the results. Even though our new additional indices related to the characteristics of scientific citations show weak correlations with citation frequency, their positive correlations also support the relationship between science and technology.

The frame we propose to study the technological frontier is open, and additional machine learning models could be used to analyze the highly cited patents in a domain and examine the relations between different variables and citation frequency. Thus, one can predict the citation frequency of new patents once published and identify the technological frontier based on potential highly cited patents. This study can help us further understand the relationships between influencing factors and the citation frequency in a domain and contributes to the intelligent identification of the technological frontier in a timely and accurate manner.

There are also some limitations in this study. First, we suggested the indices using the literature reviews, in future studies, more factors that may affect the citation frequency of patents should be introduced. Second, more machine learning models could be utilized to analyze the technological frontier, and we could select the model with high classification accuracy. In addition, to clarify the different factors that affect the citation frequency of patents in different domains, we would like to conduct further analysis of various technological domains, especially the domains with significantly different technological knowledge.

## Supporting information

S1 File The patent data of EV domain. (XLSX)
